# Carbon-Doping as Efficient Strategy for Improving Photocatalytic Activity of Polysilicon Supported Pd in Hydrogen Evolution from Formic Acid

**DOI:** 10.3390/polym13223919

**Published:** 2021-11-12

**Authors:** Amal Al-Azmi, Sajjad Keshipour

**Affiliations:** 1 Chemistry Department, Kuwait University, P.O. Box 5969, Safat 13060, Kuwait; 2 Department of Nanotechnology, Faculty of Science, Urmia University, P.O. Box 165, Urmia 5756151818, Iran; s.keshipour@urmia.ac.ir

**Keywords:** polymer, nanostructures, silicon, hydrogen generation, palladium

## Abstract

Interest in cost-effective materials pushes researchers to the inexpensive and abundant semiconductors to use photons’ energy for generating electrons and holes required for photocatalytic transformations. At the same time, polysilicon is one of the economic semiconductors with a disadvantage of high bandgap which could be solved by carbon-doping. We employed this strategy to the synthesis of carbon-doped polysilicon by a new approach starting from citric acid and methyltrimethoxysilane. The nanocomposite obtained was utterly characterized, and compared with bare polysilicon; increased UV–Vis absorbance and shift to higher wavelengths were the most notable characteristics of the synthesized catalyst. The carbon-doped polysilicon was modified with Pd nanoparticles to obtain a new heterogeneous photocatalyst for the formic acid degradation. The decomposition of formic acid was photocatalyzed by the obtained nanocomposite with a hydrogen production turnover frequency of up to 690 h^−1^. Moreover, it was demonstrated that the catalyst is stable and recyclable.

## 1. Introduction

In recent years, remarkable progress in the energy transition from fossil fuels to green alternatives such as hydrogen, whose combustion product is simply water, has taken place. Big companies are looking for the efficient production of hydrogen because they are developing future engines based on clean fuels with high energy potential. Thus, there is a significant research focus to obtain the most effective approach for the hydrogen generation. Researchers emphasize developing catalysts with superior efficiency, stability, and selectivity for efficient hydrogen production [1]. In this regard, transition metals such as Pt, Pd, Ru, Cu, and Co have been used in combination with a wide variety of organic and mineral compounds [2]. The Pd has been employed as an active catalyst for hydrogen generation combined with organic supports such as carbon-based materials [3]. While most of the Pd-catalyzed hydrogen generation reactions are focused on water splitting [4], Pd-based catalysts also efficiently promoted the degradation of formic acid (FA) [5], hydrazine [6], and amino borane [7]. Moreover, Pd perched on semiconductors can catalyze hydrogen evolution reactions through photocatalytic routes [8].

Semiconductors are widely employed in various applications on account of their compactness, inexpensiveness, reliability, and power efficiency. Polysilicon (PS)-based semiconductors are potentially active species for optical proposes, especially when doped by an atom such as B or N [9]. The C-doped PS is one of the less-studied species [10,11,12,13,14,15,16]. The large bandgap of PS requires the photocatalytic reactions by this compound to be conducted under highly energetic ultraviolet (UV) rays; this problem could be avoided by doping PS with C [17]. While this abundant and non-toxic semiconductor appears to be an excellent support for photocatalysts, photocatalytic studies employing C-doped PS as the support are rare. The C-doped SiO_2_ promotes the photocatalytic degradation of Rhodamine B and methyl orange under visible light [18]. Furthermore, in 2010, Xue et al. reported a significant increase in the photoconductivity of Pd-doped PS [19]. These positive effects of C and Pd atoms on the photo-activity of silicon-based semiconductors encouraged us to evaluate the simultaneous effects of these atoms on PS in the photocatalytic degradation of FA for hydrogen generation.

The catalytic degradation of FA was carried out using various catalysts, among which Pd-based catalysts are of particular importance on account of their superior activity. The Pd nanocrystals [20], Pd supported on carbon [21], and Pd nanoparticles supported on diamine-alkalised reduced graphene oxide [22] are some successful examples of catalysts used for the FA degradation reaction. This active catalyst showed high conversion for the FA degradation reaction especially when used as an alloy with Ag [23], Au [24], Cu [25], Co [26], Ni [27], Pt [28], Cr [29], Sn [30], Zn [31], and Ru [32]. Moreover, Pd supported on Si-based supports has been employed as the catalyst for this reaction, such as amine-functionalized SBA-15 [33,34], dithiocarbamate-functionalized SBA-15 [35], mesoporous silica supported Pd−MnO_x_ [36], and silica nanosphere [37].

**Scheme 1:**
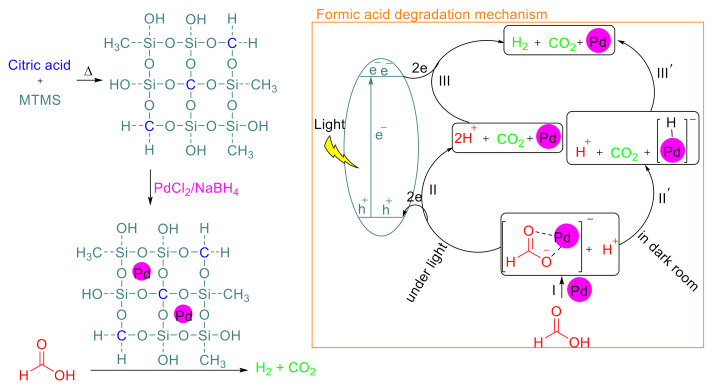
Formic acid degradation by Pd@C–PS and the proposed reaction mechanism.

In our continued efforts to develop efficient catalytic systems [38,39,40,41], herein, we tried to utilize PS as a support for Pd nanoparticles to synthesis a new photocatalyst (Scheme 1). To transform PS into an efficient semiconductor for the powerful light absorption, doping of the polymer was carried out by C via a new procedure, including thermal treatment of methyltrimethoxysilane (MTMS) in the presence of citric acid (CA). The CA is an active starting material for the constructing C-based materials, in particular, when it is thermally treated [42]. This strategy would be a promising pathway for the synthesis of optically active support for the Pd nanoparticles to obtain a novel heterogeneous photocatalyst for formic acid degradation. Considering that the Pd tends to create complexes with formate ligands, we believe that the formation of the Pd-formate complex is inevitable (the path I, Scheme 1). The formed complex provides CO_2_ and H^+^ with losing two electrons (the path II, Scheme 1) [43], which the produced protons subsequently evolved to H_2_ by taking up the two electrons in the presence of Pd (path III, Scheme 1) [44]. However, it was shown that the reaction has proceeded in the absence of light in three steps, including: (a) the formation of Pd-formate, (b) releasing CO_2_ from Pd-formate, and formation of Pd-hydride, and (c) finally the reaction of hydride by a proton to produce H_2_ [45].

## 2. Experimental

### 2.1. Preparation of Pd@C-PS

In a beaker, 2 g of CA and 5 mL of MTMS were mixed and placed on a hot plate and heated to 200 °C. The mixture started to bubble and solidify, and a white solid formed after 10 min. Subsequently, the hot plate was turned off and 30 mL of deionized water was added to the mixture. The solid was suspended using a spatula, and the mixture was sonicated for 15 min; then, it was filtered. The residue was washed twice with 50 mL of deionized water and 20 mL of acetone to afford 3.1 g of white solid; C–PS. 0.04 g of PdCl_2_ was sonicated in 10 mL of deionized water and added to 2 g of C–PS in 10 mL H_2_O, and the resulting mixture was stirred at room temperature for 6 h. Then, 0.1 M NaBH_4_ was added to the obtained mixture at a rate of 2 drops per min till the reaction mixture stopped bubbling. An excess of 2 mL of NaBH_4_ was added to ensure that all the Pd(II) was reduced to Pd nanoparticles. Finally, the light grey solid was separated by filtration, washed with 20 mL of deionized water five times, and dried in an oven at 80 °C to obtain 1.91 g of the final catalyst Pd@C–PS.

### 2.2. Determination of Pd on Pd@C-PS Using Flame AAS

To 10 mL of HCl:HNO_3_ (3:1) mixture, 0.3 g of Pd@C–PS was added, and the mixture was sonicated for 3 h. Next, the total volume of the filtrate was increased to 20 mL by diluting it with distilled water. The final solution was aspirated into the AAS flame against the blank prepared with C–PS. The Pd concentration was obtained using a calibration curve prepared using Pd solution standards.

### 2.3. Typical Procedure for the FA Degradation

A two-necked round-bottom flask was placed in a box equipped with a 300 W Xenon lamp and a hose connected one of the necks to a 12 M NaOH trap. Another hose connected the trap to a gas burette. To the flask, 30 mL of FA and 0.05 g of the catalyst were added from the other neck; then, the neck was closed using a rubber stopper. Subsequently, the lamp was turned on and the mixture was mechanically stirred. The bubbling of the generated gases was initiated in the gas burette, and the volume changes were recorded every 10 min by measuring the replaced water by gasses. The TOF was obtained by the following equation:

TOF (h^−1^) = (PV)/(2R·T·n_Au_·t)

where P is atmospheric pressure (atm), V is the volume of produced gas (mL), R is the universal gas constant (0.08206 L·atm/mol·K), T is the temperature (K), n_Au_ is the mmol of Au in the used catalyst, and t is time (h).

## 3. Results and Discussion

### 3.1. Characterization of the Synthesized Materials

**Figure 1:**
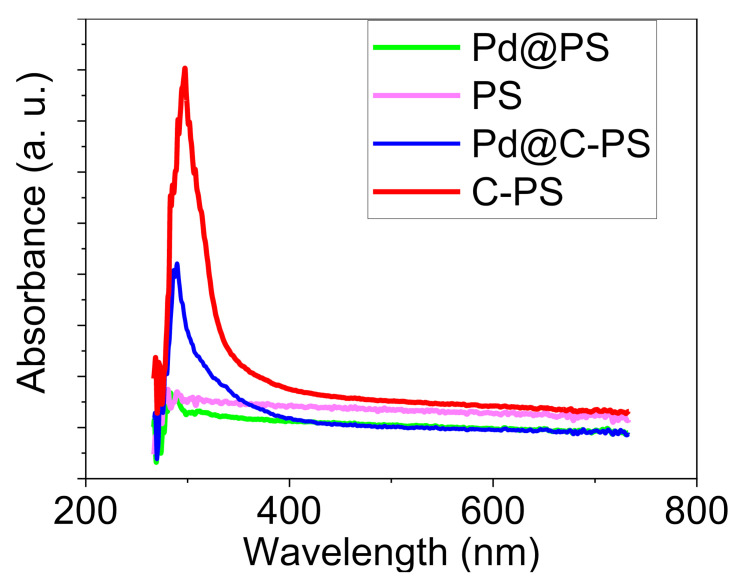
UV–Vis spectra of PS, C–PS, Pd@PS, and Pd@C–PS.

Considering that our primary purpose of doping PS with C-atoms was to improve the optical properties, the UV–Vis spectra of PS, C–PS, Pd@PS, and Pd@C–PS were first investigated (Figure 1). Although PS showed weak absorbance at 227 nm, C–PS indicated more substantial absorbance at 245 nm at the same concentration. In addition, the observed 18 nm band shift indicates that the C-doping into PS leads to a shift to higher wavelengths [17]. Moreover, the deposition of Pd decreased the absorbance wavelengths in both PS and C–PS to 219 and 236 nm, respectively. These increasing in absorption and shifts to higher wavelengths show that Pd@C–PS could be used as an efficient photocatalyst. The bandgap energies of PS, C-PS, Pd@PS, and Pd@C–PS are 5.45, 5.05, 5.65, and 5.24 eV, respectively. As a matter of fact, a new photocatalyst of Pd supported on silicon material, Pd@C–PS, was synthesized, in which the catalyst is optically active as an additional advantage to the previous similar catalysts mentioned above [33,34,35,36,37]. Therefore, Pd@C–PS might promote Pd-catalyzed reactions photocatalytically in milder reaction conditions with higher efficiency compared to previous reports due to the use of photons’ energies by the semiconductor support.

**Figure 2:**
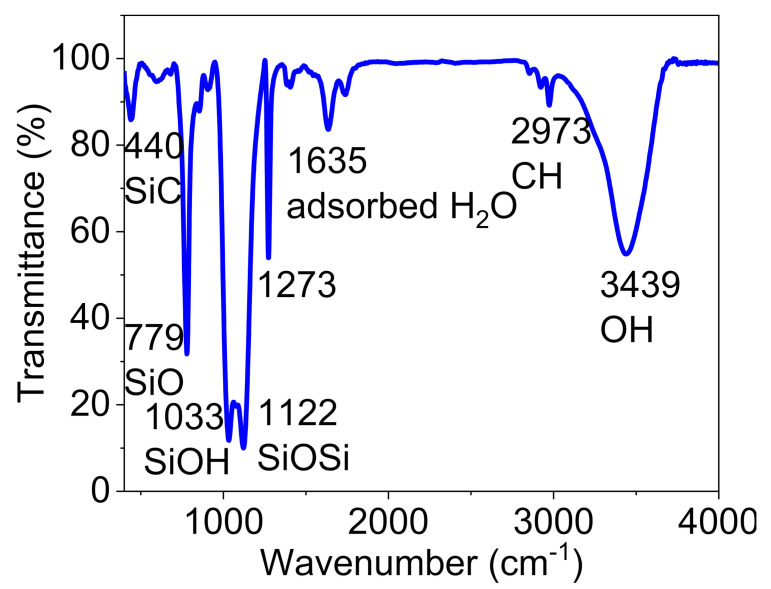
FT-IR spectrum of Pd@C–PS.

A Fourier-transform infrared (FT-IR) spectrum was prepared to characterize the functional groups of Pd@C–PS (Figure 2). The structure of the composite was approved based on the absorption bands noted at 3439, 1122, 1033, and 779 cm^−1^ for OH, Si–O–Si, Si–OH, and Si–O bonds, respectively. The appearance of two peaks at 2973 and 440 cm^−1^ confirmed the existence of carbon atoms in the composite structure, and these peaks were assigned to CH and SiC bonds, respectively [46].

**Figure 3:**
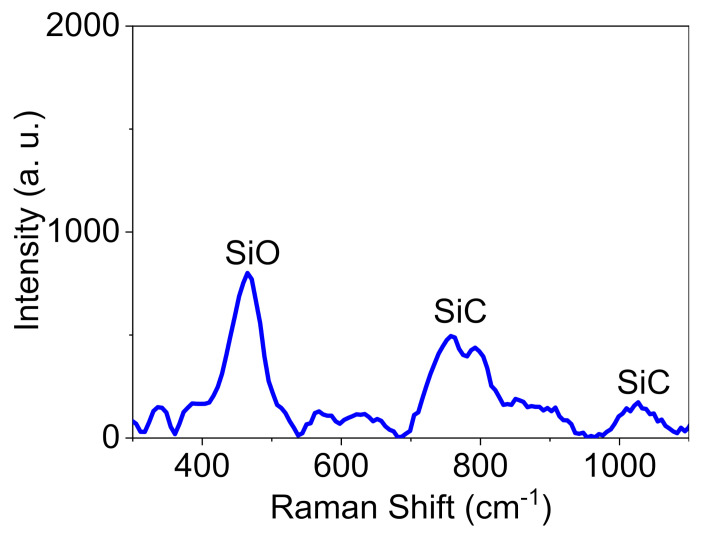
Raman spectrum of Pd@C–PS.

The Raman spectrum of Pd@C–PS also revealed peaks attributing to Si and SiC (Figure 3). The exact value of the Raman shift for Si in the composite is size-dependent and lies in the range of 470–520 cm^−^^1^ [47]. The appearance of the Si peak at about 470 cm^−1^ revealed that the sample contains particles with the size of about 10 nm. Moreover, Raman spectroscopy is considered a reliable method to assign SiC considering the appearance of peaks at around 760 and 1000 cm^−1^, which correspond to the transverse and longitudinal optical phonon modes, respectively [47].

**Figure 4:**
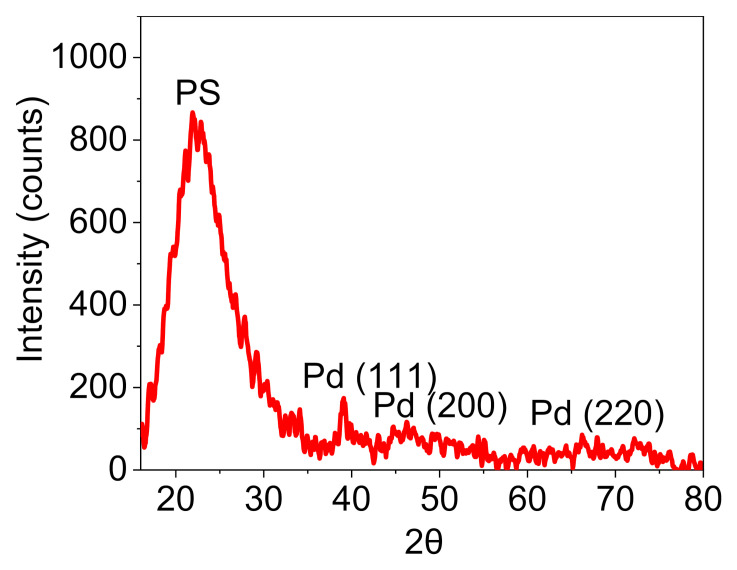
XRD pattern of Pd@C–PS.

The X-ray diffraction (XRD) pattern of Pd@C–PS was prepared to determine diffraction peaks corresponding to Si and Pd. The peak of PS at 2θ = ~22.45 and peaks of Pd corresponding to (111), (200), and (220) verified the support structure and Pd doping (Figure 4) [39].

**Figure 5:**
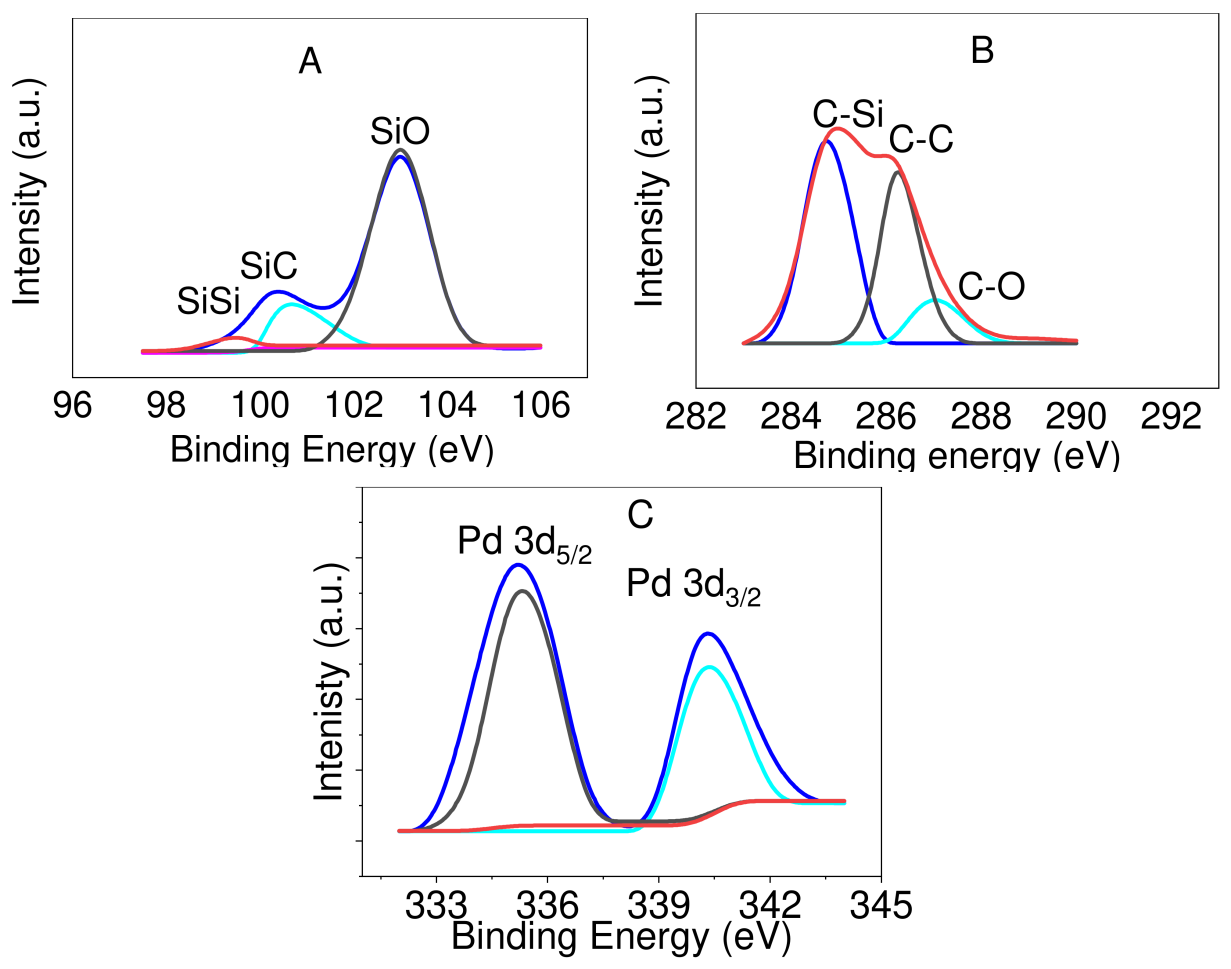
Deconvoluted XPS spectra of Pd@C–PS for the peaks ascribed to Si (**A**), C (**B**), and Pd (**C**).

The X-ray photoelectron spectrum (XPS) of Pd@C–PS was obtained to determine all the atoms present in the structure and highlight specific details about the structure, such as the Pd oxidation state and Si bonds (see ESI). The XPS showed peaks corresponding to Si, C, Pd, and O, which authenticated the proposed structure. The peaks attributed to Si 2p, C 1s, and Pd 3d are shown in Figure 5. Three peaks appeared at 99.7, 100.3, and 103.1 eV and attributed to the Si–Si, Si–C, and Si–O bonds, respectively [10]. In addition, three peaks were observed for C 1s at 284.6, 286.1, and 290.0 eV, which ascribed to the C–C, C–O, and C–Si bonds, respectively [10]. The existence of C–C peaks demonstrated that the interconnected carbon atoms in CA remained unchanged. The XPS showed peaks of Pd 3d_5/2_ at 335.5 and Pd 3d_3/2_ at 340.5 eV, which confirmed the formation of nanoparticles [39]. In addition, energy dispersive X-Ray analysis showed the loading of C and Pd on the composite (see ESI). This analysis provided the weight percentages of O, C, Si, and Pd as 40.07%, 31.03%, 27.94%, and 0.97%, respectively. The mole of Pd was calculated to be 0.09 mmol per 1 g of the catalyst.

**Figure 6:**
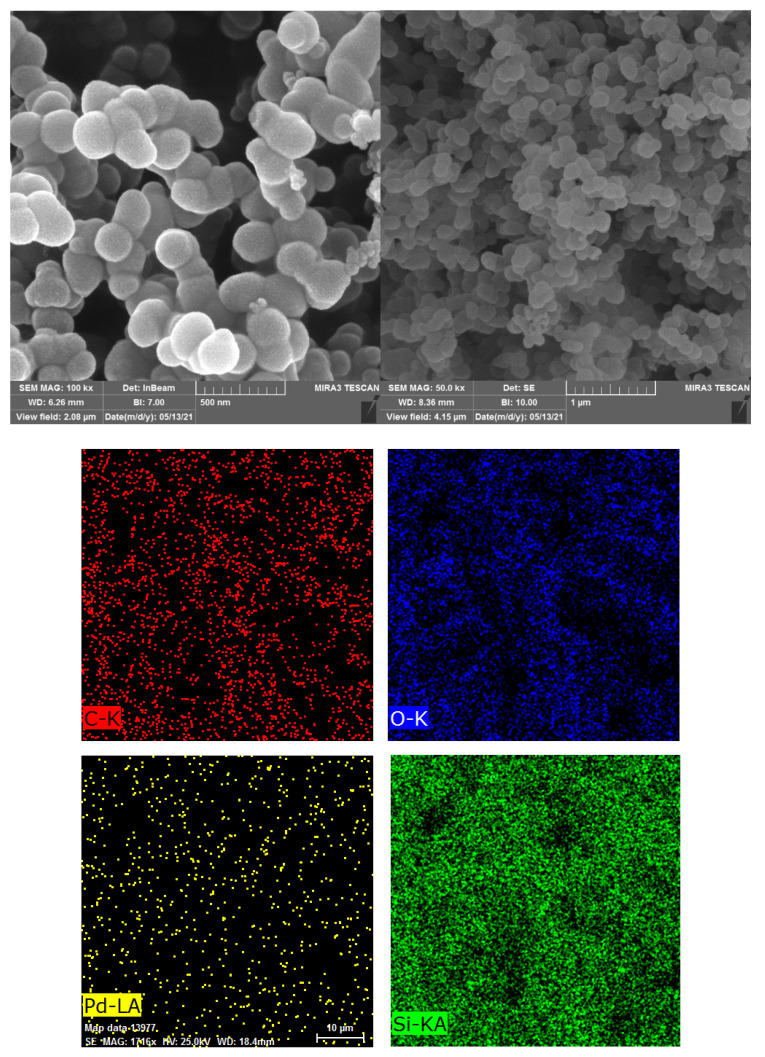
Scanning electron microscopy and elemental mapping of Pd@C–PS.

A microscopic study was conducted on Pd@C–PS to observe the surface topography, size, and shape distribution of the prepared particles (Figure 6). The obtained SEM images showed several uniform spherical particles, which were considered to be PS particles [47]. Elemental mapping was also carried for Pd@C–PS to observe the distribution of C and Pd atoms in the composite (Figure 6). This showed that the C atoms were uniformly dispersed on PS and that the C-doping improved the optical properties of PS; thus, a homogeneous support was obtained that was desirable for photo-induced reactions. Further, homogeneous distribution of Pd was observed in this analysis, indicating that a uniform catalyst was synthesized.

**Figure 7:**
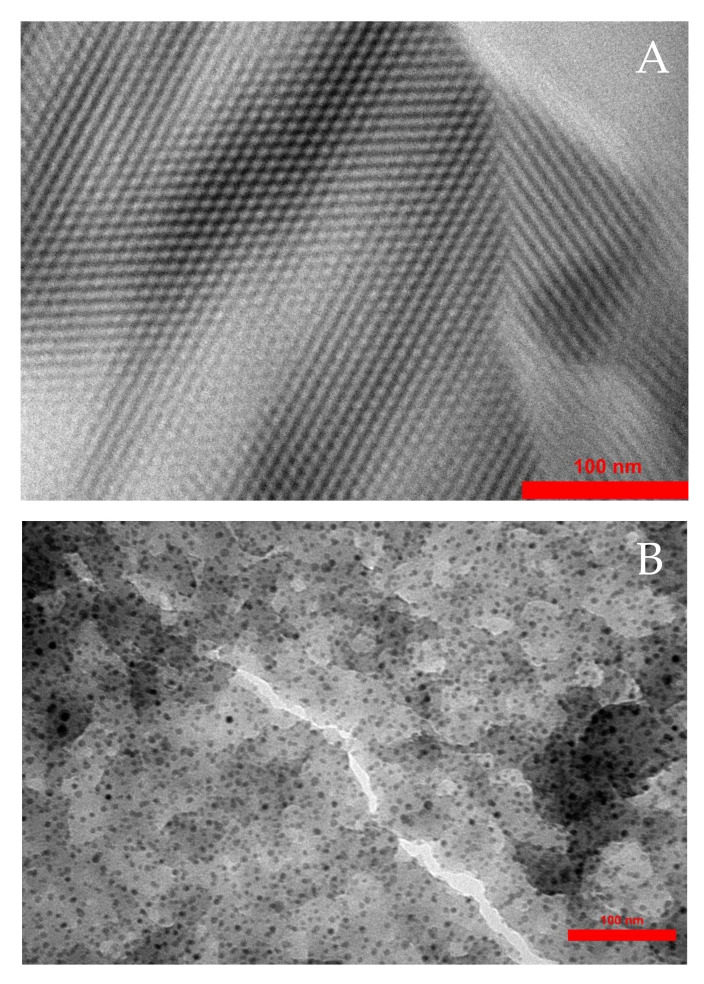
Transmission electron microscopy micrographs of C–PS (**A**), and Pd@C–PS (**B**).

The decoration of Pd nanoparticles on the composite encouraged us to focus on obtaining higher resolution images of the produced structure using transmission electron microscopy (TEM) of C–PS and Pd@C–PS (Figure 7). The TEM micrograph of C–PS indicated a homogeneous mesoporous structure, which is consistent with previous reports [10]. To elucidate the deposition of Pd nanoparticles, a TEM micrograph of Pd@C–PS was also obtained, which showed that particles other than PS are present. These particles showed a different mass-thickness contrast than the other particles, confirming the existence of particles other than PS particles with an average size of 8–12 nm.

**Figure 8:**
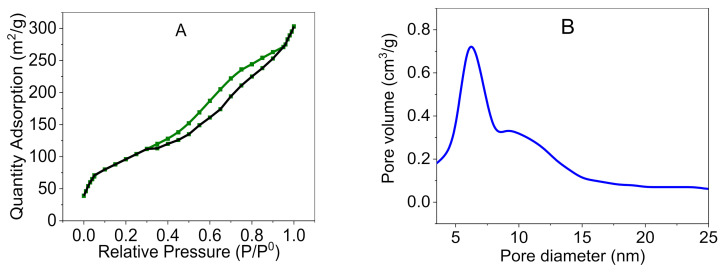
Nitrogen adsorption and desorption isotherms (**A**), and pore size distribution of Pd@C–PS (**B**).

A Brunauer–Emmett–Teller analysis of Pd@C–PS provided valuable information about the structure (Figure 8). The adsorption and desorption isotherms at 77 K revealed a type II curve, indicating a mesoporous structure with a pore size of 2–50 nm. Moreover, the presence of some hysteresis in the adsorption and desorption isotherms demonstrated that the small amount of pore-blocking might be due to the presence of Pd nanoparticles [48]. The specific surface area was calculated to be 283 m^2^/g. Furthermore, the pore size distribution revealed a maximum size of 6.10 nm of the composite.

### 3.2. Investigation of the Photocatalytic Activity of Pd@C-PS

A series of circumstances is required for efficient hydrogen generation from FA degradation under light irradiation. Photocatalysts having high catalytic activity, superior absorbance in the desired optical region, and the possibility of recovery and recyclability meet these needs. Although numerous catalysts have already been developed for FA degradation, a powerful one has not yet been found. The high catalytic activity of Pd for FA degradation, satisfying absorbance of the UV–Vis light of the prepared composite and heterogeneity of Pd@C–PS in the FA solution makes the prepared composite a promising catalyst for hydrogen generation. Therefore, the photocatalytic degradation of FA catalyzed by Pd@C–PS under light irradiation and effects of various parameters and conditions have been investigated.

**Figure 9:**
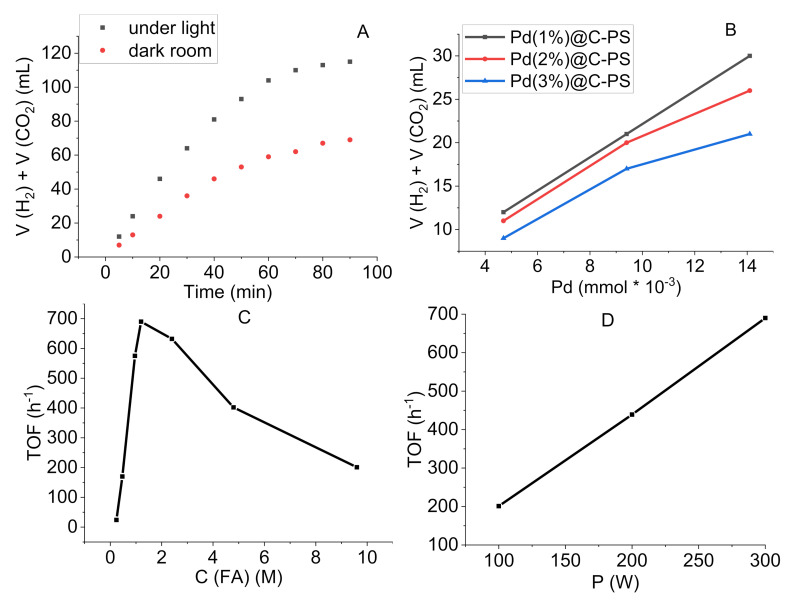
FA degradation toward H_2_/CO_2_ production: (**A**) Volume of the produced gases (H_2_ + CO_2_) as a function of reaction time, (**B**) Volume of the produced gases (H_2_ + CO_2_) as a function of Pd concentration at 10 min, (**C**) Catalytic TOF of the reaction as a function of FA concentration at 10 min, and (**D**) Catalytic TOF of the reaction as a function of light power at 10 min.

Strong evidence of the catalytic activity of Pd was confirmed when the reaction was conducted using PS and C–PS (without Pd doping), in which none of them promoted the degradation reaction. Further assays using Pd@C–PS and Pd@PS demonstrated the benefits of C-doping. As expected, C-doping enhanced the photo absorbance of the composite, increasing the efficiency of Pd@C–PS in the decomposition reaction compared to that of Pd@PS. As a result, 0.53 mmol of hydrogen was obtained in 10 min by Pd@C–PS vs. 0.41 mmol of hydrogen generated by Pd@PS. Furthermore, all kinds of the employed catalysts showed lower yields in a dark room, which confirmed the photo-induced pathway (Figure 9A). For instance, Pd@C–PS produced 0.53 mmol and 0.29 mmol of hydrogen in 10 min under light irradiation and in a dark room, respectively. A significant positive correlation between the Pd loading and hydrogen generated when using Pd@C–PS was noted (Figure 9B). In this study, three kinds of catalysts, including Pd (1 w%)@C–PS, Pd (2 w%)@C–PS, and Pd (3 w%)@C–PS were examined. The Pd (1 w%)@C–PS showed better performance compared to the others, indicating that the lower concentration and broader distribution of Pd provides a more efficient catalyst. Various concentrations of FA were screened to find the optimal reactant concentration (Figure 9C); 0.12 M of FA afforded the highest turnover frequency (TOF) of 690 h^−1^ (calculated in 10 min). Surprisingly, the power of light irradiation obtained by the lamp used had an impressive effect on the reaction yield with higher power affording higher conversion (Figure 9D).

**Figure 10:**
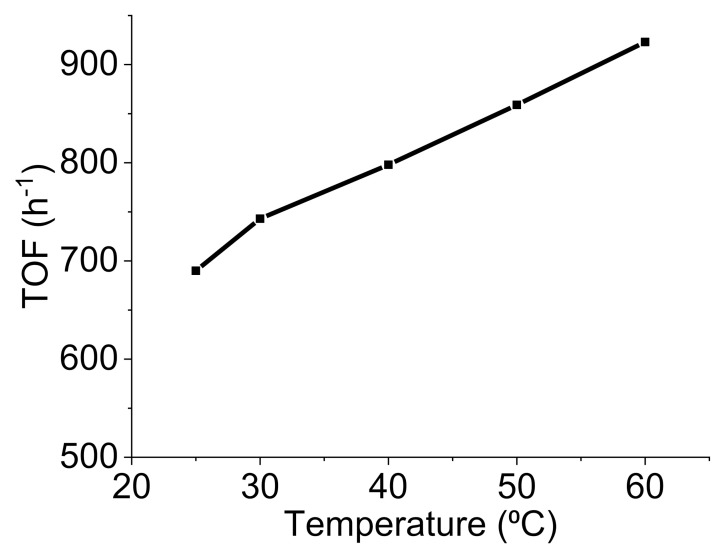
The temperature effect on TOF of FA degradation by Pd@C–PS.

In addition, the effect of the reaction temperature on the reaction rate was evaluated to determine kinetic parameters such as the activation energy (E_a_). The reaction was carried out at 19, 25, 30, 40, 50, and 60 °C (Figure 10). Interestingly, the reaction rate increased with temperature, as shown in Figure 10. The E_a_ was calculated to be 3.6 KJ/mol for the reaction catalyzed by Pd@C–PS.

**Figure 11:**
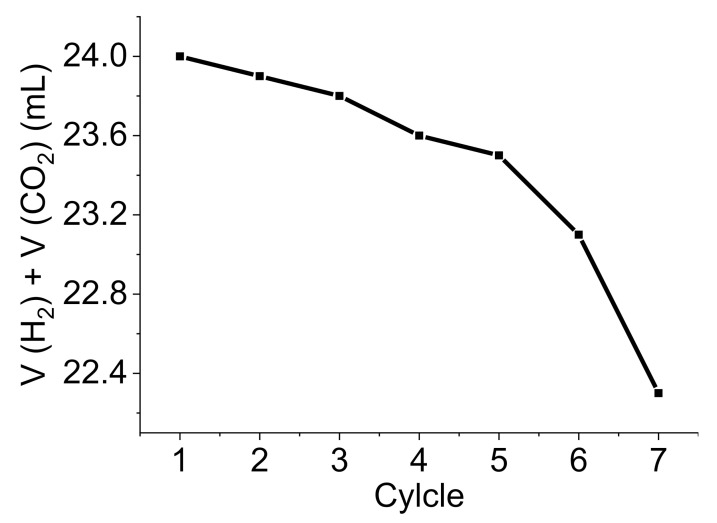
Recyclability test of Pd@C–PS for the FA degradation towards H_2_/CO_2_ production.

Stability is one of the notable characteristics of heterogeneous catalysts because it offers recyclability. This could be evaluated by conducting a catalyst leaching study, which shows the catalyst releasing to the reaction media. Therefore, the amount of Pd released from Pd@C–PS into the FA solution of the degradation reaction was investigated using flame atomic absorption spectroscopy (AAS). The catalyst was filtered off, and the filtrate was analyzed to determine the Pd concentration using calibration curves obtained from various concentrations of Pd. Interestingly, the Pd concentration was lower than the detection limit of the instrument (i.e., less than 9 µg/L), indicating that Pd@C–PS is relatively stable. Moreover, a hot filtration test was conducted to check the reaction phase, in which Pd@C–PS was separated by filtration after 15 min from the start of the reaction, and the reaction progress was monitored. The reaction quenching in this stage approved the heterogeneous progressing of it. Finally, the Pd@C–PS recyclability was examined for the FA degradation reaction. The Pd@C–PS was separated and reused for the degradation reaction for seven cycles. No significant decrease in the produced gas volumes was noted (Figure 11). Furthermore, the FT-IR spectrum of the recovered catalyst revealed no significant changes in the main peaks (see ESI).

Finally, the TOF of the FA degradation catalyzed by Pd@C–PS was compared with those from previous reports that used amine-functionalized SBA-15 [33,34], dithiocarbamate-functionalized SBA-15 [35], mesoporous silica supported Pd-MnO_x_ [36], and silica nanospheres as the catalyst for FA degradation [37]. While some of these catalysts showed better performances than Pd@C–PS without photo-irradiation, our catalyst showed satisfying activity under photocatalytic conditions (see ESI). The better the performance of Pd@C–PS compared to some of the reports listed here is attributed to the promoting of the Pd-catalyzed evolution reaction by the driving force of C–PS as the semiconductor, generating electrons and holes under irradiation, which in turn accelerate redox transformations. Promoting the reaction by Pd@C–PS with low yield in the darkroom (Figure 9A) demonstrated the importance of electron/hole creation by light in the reaction mixture. In addition, a better performance of Pd@C–PS in the degradation reaction compared to the C-doped SiO_2_ is ascribed to the presence of Pd as a powerful catalyst [18]. This study could create a new insight into the application of silicon-based heterogeneous catalysts since the catalyst activity was enhanced remarkably with C-doping as a bit of manipulation in the structure.

## 4. Conclusions

In conclusion, a new composite of Pd was fabricated in two distinct stages, including the preparation of C-doped PS via the thermal treatment of a mixture of PS precursor/CA, and subsequent deposition of Pd nanoparticles on the support. A UV−Vis absorbance study was conducted for the composite, which indicated enhanced optical behaviors, confirming its potential to be employed in photocatalytic reactions. After complete characterization, the composite employed in the FA degradation reaction showed a TOF of up to 690 h^−1^. The reaction yield increased with temperature, amount of Pd loading, FA concentration, and irradiation power. Moreover, the recycling study confirmed that the catalyst has high stability and can be recycled several times. In our view, this work gives a new perspective on the utilization of photocatalysts based on C–PS for various chemical reactions. Future works should concentrate on the deposition of other transition metals on C–PS to assay the possibility of synthesizing potent photocatalysts. In addition, C-doping in PS needs more studies to be optimized by introducing new pathways.

## Data Availability

The data presented in this study are available in the article and Supplementary Materials.

## Supplementary Materials

The following are available online at https://www.mdpi.com/article/10.3390/polym13223919/s1, Figure S1: EDX analysis of Pd@C-PS, Figure S2: XPS analysis of Pd@C-PS, Figure S3: FT-IR analysis of recovered Pd@C-PS, Figure S4: GC spectrum of the produced gas mixture, Table S1: Comparison of TOFs obtained by various catalysts for FA degradation.

## References

[1] Othman M.F., Adam A., Najafi G., Mamat R. (2017). Green fuel as alternative fuel for diesel engine: A review. Renew. Sustain. Energy Rev..

[2] Acar C., Dincer I. (2019). Review and evaluation of hydrogen production options for better environment. J. Clean. Prod..

[3] Cardoso J.A.S.B., Šljukić B., Erdem M., Sequeira C.A.C., Santos D.M.F. (2018). Vine Shoots and Grape Stalks as Carbon Sources for Hydrogen Evolution Reaction Electrocatalyst Supports. Catalysts.

[4] Alex C., Bhat S.A., John N.S., Yelamaggad C.V. (2019). Highly Efficient and Sustained Electrochemical Hydrogen Evolution by Embedded Pd-Nanoparticles on a Coordination Polymer—Reduced Graphene Oxide Composite. ACS Appl. Energy Mater..

[5] Wang Z.-L., Yan J.-M., Wang H.-L., Ping Y., Jiang Q. (2013). Au@Pd core–shell nanoclusters growing on nitrogen-doped mildly reduced graphene oxide with enhanced catalytic performance for hydrogen generation from formic acid. J. Mater. Chem. A.

[6] Chen J., Mei L., Liu J., Zhong C., Yuan B., Li Q. (2019). Microwave-assisted iodine-catalyzed oxidative coupling of dibenzyl(difurfuryl)disulfides with amines: A rapid and efficient protocol for thioamides. RSC Adv..

[7] Jia H., Zhu Y., Song X., Zheng X., Liu P. (2018). Magnetic graphene oxide-ionic liquid grafted chitosan composites anchored Pd(0) nanoparticles: A robust heterogeneous catalyst with enhanced activity and superior reusability for hydrogen generation from ammonia borane. Int. J. Hydrogen Energy.

[8] Sayed F.N., Sasikala R., Jayakumar O.D., Rao R., Betty C.A., Chokkalingam A., Kadam R.M., Jagannath J., Bharadwaj S.R., Vinu A. (2014). Photocatalytic hydrogen generation from water using a hybrid of graphene nanoplatelets and self doped TiO2–Pd. RSC Adv..

[9] Li N., Xiang F., Fratalocchi A. (2021). Silicon-Based Photocatalysis for Green Chemical Fuels and Carbon Negative Technologies. Adv. Sustain. Syst..

[10] Zhang Y., Chen H., Wang S., Shao W., Qin W., Zhao X., Kong F. (2020). Facile fabrication and structure control of SiO2/carbon via in situ doping from liquefied bio-based sawdust for supercapacitor applications. Ind. Crop. Prod..

[11] Han L.M., Pan J.-S., Chen S.-M., Balasubramanian N., Shi J., Wong L.S., Foo P.D. (2001). Characterization of Carbon-Doped SiO2 Low k Thin Films: Preparation by Plasma-Enhanced Chemical Vapor Deposition from Tetramethylsilane. J. Electrochem. Soc..

[12] Sikder A.K., Kumar A., Thagella S., Yota J. (2004). Effects of Properties and Growth Parameters of Doped and Undoped Silicon Oxide Films on Wear Behavior During Chemical Mechanical Planarization Process. J. Mater. Res..

[13] Lubguban J., Kurata Y., Inokuma T., Hasegawa S. (2000). Thermal stability and breakdown strength of carbon-doped SiO2:F films prepared by plasma-enhanced chemical vapor deposition method. J. Appl. Phys..

[14] Ma L., Wang Y., Wang Y., Fu S. (2019). Formation of C-doped SiO2 coatings on carbon fibers by the sol-dipping process. Colloids Surf. A Physicochem. Eng. Asp..

[15] Zhao L., Barbarin Y., Croes K., Baklanov M.R., Verdonck P., Tőkei Z., Claeys C. (2015). Impact of carbon-doping on time dependent dielectric breakdown of SiO2-based films. Appl. Phys. Lett..

[16] van Druenen M., Collins G., Glynn C., O’Dwyer C., Holmes J.D. (2018). Functionalization of SiO2 Surfaces for Si Monolayer Doping with Minimal Carbon Contamination. ACS Appl. Mater. Interfaces.

[17] Mungondori H.H., Tichagwa L. (2012). Photo-Catalytic Activity of Carbon/Nitrogen Doped TiO2-SiO2 under UV and Visible Light Irradiation. Mater. Sci. Forum.

[18] Zhang D., Wu J., Zhou B., Hong Y., Li S., Wen W. (2013). Efficient photocatalytic activity with carbon-doped SiO2 nanoparticles. Nanoscale.

[19] Xue Q., Ma M., Zhen Y., Zhou X., Wang S. (2012). Large photoconductivity of Pd doped amorphous carbon film/SiO2/Si. Diam. Relat. Mater..

[20] Shao M., Odell J., Humbert M., Yu T., Xia Y. (2013). Electrocatalysis on Shape-Controlled Palladium Nanocrystals: Oxygen Reduction Reaction and Formic Acid Oxidation. J. Phys. Chem. C.

[21] Bulushev D.A., Beloshapkin S., Ross J.R.H. (2010). Hydrogen from formic acid decomposition over Pd and Au catalysts. Catal. Today.

[22] Eppinger J., Huang K.-W. (2017). Formic Acid as a Hydrogen Energy Carrier. ACS Energy Lett..

[23] Choi B.-S., Song J., Song M., Goo B.S., Lee Y.W., Kim Y., Yang H., Han S.W. (2019). Core–Shell Engineering of Pd–Ag Bimetallic Catalysts for Efficient Hydrogen Production from Formic Acid Decomposition. ACS Catal..

[24] Sahoo D.P., Patnaik S., Rath D., Mohapatra P., Mohanty A., Parida K. (2019). Influence of Au/Pd alloy on an amine functionalised ZnCr LDH–MCM-41 nanocomposite: A visible light sensitive photocatalyst towards one-pot imine synthesis. Catal. Sci. Technol..

[25] Li G., Kobayashi H., Kusada K., Taylor J.M., Kubota Y., Kato K., Takata M., Yamamoto T., Matsumura S., Kitagawa H. (2014). An ordered bcc CuPd nanoalloy synthesised via the thermal decomposition of Pd nanoparticles covered with a metal–organic framework under hydrogen gas. Chem. Commun..

[26] Wang Z.-L., Yan J.-M., Ping Y., Wang H.-L., Zheng W.-T., Jiang Q. (2013). An Efficient CoAuPd/C Catalyst for Hydrogen Generation from Formic Acid at Room Temperature. Angew. Chem. Int. Ed..

[27] Yurderi M., Bulut A., Zahmakiran M., Kaya M. (2014). Carbon supported trimetallic PdNiAg nanoparticles as highly active, selective and reusable catalyst in the formic acid decomposition. Appl. Catal. B Environ..

[28] Arenz M., Stamenkovic V., Schmidt T.J., Wandelt K., Ross P.N., Markovic N.M. (2003). The electro-oxidation of formic acid on Pt–Pd single crystal bimetallic surfaces. Phys. Chem. Chem. Phys..

[29] Yurderi M., Bulut A., Caner N., Celebi M., Kaya M., Zahmakiran M. (2015). Amine grafted silica supported CrAuPd alloy nanoparticles: Superb heterogeneous catalysts for the room temperature dehydrogenation of formic acid. Chem. Commun..

[30] Colmati F., Antolini E., Gonzalez E.R. (2005). Pt–Sn/C electrocatalysts for methanol oxidation synthesized by reduction with formic acid. Electrochim. Acta.

[31] Jeroro E., Vohs J.M. (2009). Reaction of Formic Acid on Zn-Modified Pd(111). Catal. Lett..

[32] Choi J.-H., Jeong K.J., Dong Y., Han J., Lim T., Lee J.-S., Sung Y. (2006). Electro-oxidation of methanol and formic acid on PtRu and PtAu for direct liquid fuel cells. J. Power Sources.

[33] Koh K., Jeon M., Yoon C.W., Asefa T. (2017). Formic acid dehydrogenation over Pd NPs supported on amine-functionalized SBA-15 catalysts: Structure–activity relationships. J. Mater. Chem. A.

[34] Jin M.-H., Park J.-H., Oh D., Park J.-S., Lee K.-Y., Lee D.-W. (2019). Effect of the amine group content on catalytic activity and stability of mesoporous silica supported Pd catalysts for additive-free formic acid dehydrogenation at room temperature. Int. J. Hydrogen Energy.

[35] Farajzadeh M., Alamgholiloo H., Nasibipour F., Banaei R., Rostamnia S. (2020). Anchoring Pd-nanoparticles on dithiocarbamate- functionalized SBA-15 for hydrogen generation from formic acid. Sci. Rep..

[36] Jin M.-H., Oh D., Park J.-H., Lee C.-B., Lee S.-W., Park J.-S., Lee K.-Y., Lee D.-W. (2016). Mesoporous Silica Supported Pd-MnOx Catalysts with Excellent Catalytic Activity in Room-Temperature Formic Acid Decomposition. Sci. Rep..

[37] Yadav M., Singh A.K., Tsumori N., Xu Q. (2012). Palladium silica nanosphere-catalyzed decomposition of formic acid for chemical hydrogen storage. J. Mater. Chem..

[38] Al-Azmi A., Keshipour S. (2020). Dimaval as an efficient ligand for binding Ru(III) on cross-linked chitosan aerogel: Synthesis, characterisation and catalytic investigation. Cellulose.

[39] Keshipour S., Al-Azmi A. (2020). Synthesis and catalytic application of Pd/PdO/Fe3O4@polymer-like graphene quantum dots. Appl. Organomet. Chem..

[40] Al-Azmi A., Keshipour S. (2019). Cross-linked chitosan aerogel modified with Pd(II)/phthalocyanine: Synthesis, characterization, and catalytic application. Sci. Rep..

[41] Keshipour S., Mohammad-Alizadeh S. (2021). Nickel phthalocyanine@graphene oxide/TiO2 as an efficient degradation catalyst of formic acid toward hydrogen production. Sci. Rep..

[42] Ahmadi H., Keshipour S., Ahour F. (2020). New water-soluble colorimetric pH and metal ione sensor based on graphene quantum dot modified with alizarine red S. Sci. Rep..

[43] He M., Li Z.H. (2016). Palladium-atom catalyzed formic acid decomposition and the switch of reaction mechanism with temperature. Phys. Chem. Chem. Phys..

[44] Teixeira I.F., Quiroz J., Homsi M.S., Camargo P.H.C. (2020). An Overview of the Photocatalytic H2 Evolution by Semiconductor-Based Materials for Nonspecialists. J. Braz. Chem. Soc..

[45] Miriam N.G., Kohsuke M., David S.T., Yasutaka K., Hiromi Y. (2019). New Approaches Toward the Hydrogen Production From Formic Acid Dehydrogenation Over Pd-Based Heterogeneous Catalysts. Front. Mater..

[46] Li K.-M., Jiang J.-G., Tian S.-C., Chen X.-J., Yan F. (2014). Influence of Silica Types on Synthesis and Performance of Amine–Silica Hybrid Materials Used for CO2 Capture. J. Phys. Chem. C.

[47] Parida B., Choi J., Lim G., Kim K., Kim K. (2013). Enhanced Visible Light Absorption by 3C-SiC Nanoparticles Embedded in Si Solar Cells by Plasma-Enhanced Chemical Vapor Deposition. J. Nanomater..

[48] Rafigh S.M., Heidarinasab A. (2017). Mesoporous Chitosan–SiO2 Nanoparticles: Synthesis, Characterization, and CO2 Adsorption Capacity. ACS Sustain. Chem. Eng..

