# Supplementary material for: Carbon-Doping as Efficient Strategy for Improving Photocatalytic Activity of Polysilicon Supported Pd in Hydrogen Evolution from Formic Acid
Source: Polymers (Basel). 2021 Nov 12;13(22):3919. doi: 10.3390/polym13223919 (PMC8624602; doi:10.3390/polym13223919)
Supplement: Supplementary file 1 [file polymers-13-03919-s001.zip › polymers-1438532-supplementary.pdf]

Carbon doping as efficient strategy for improving photocatalytic activity of polysilicon supported Pd in hydrogen evolution from formic acid

Amal Al-Azmi,[a]* Sajjad Keshipour[b]

[a]Chemistry Department, Kuwait University, P. O. Box 5969, Safat 13060, Kuwait E-mail: amalrchem@gmail.com, amal.alazemi@ku.edu.kw.

[b]Department of Nanotechnology, Faculty of Science, Urmia University, Urmia, Iran

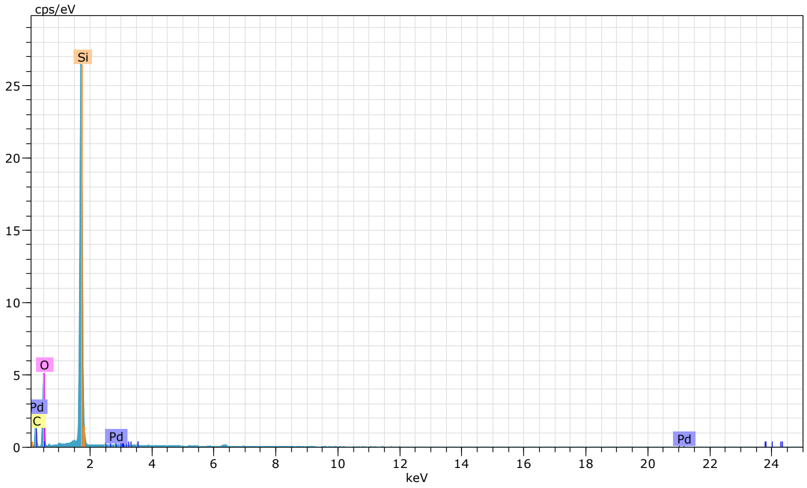

Figure S1. EDX analysis of Pd@C-PS.

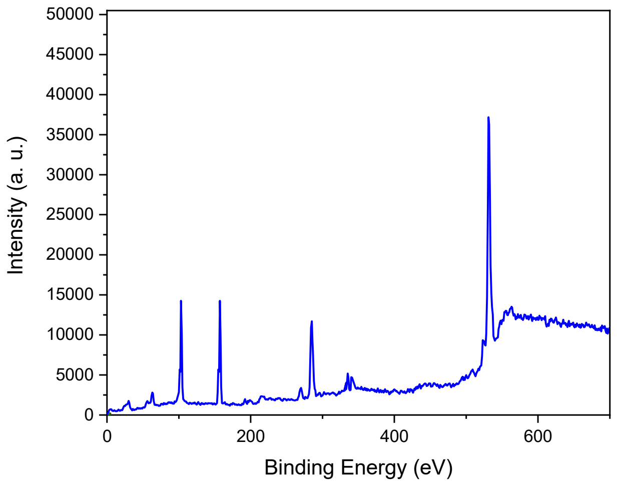

Figure S2. XPS analysis of Pd@C-PS.

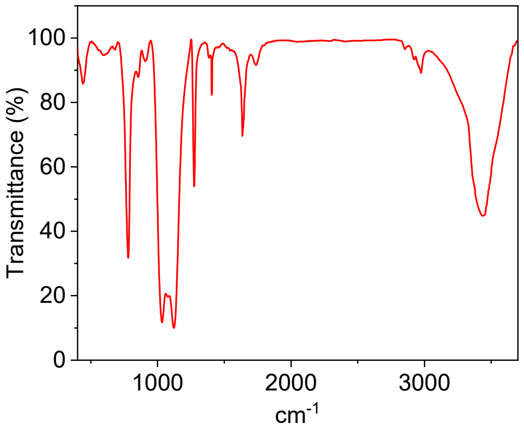

Figure S3. FT-IR analysis of recovered Pd@C-PS.

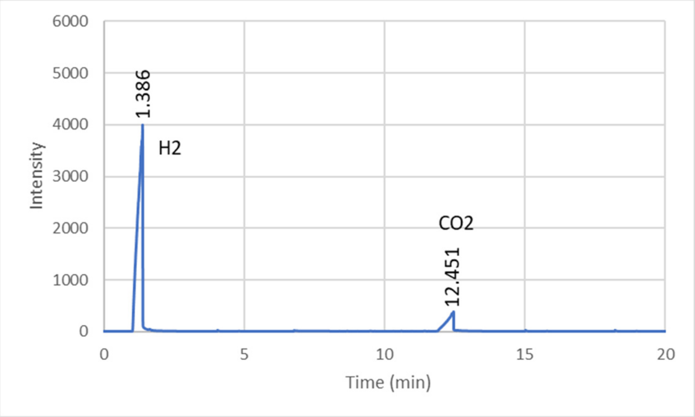

Figure S4. GC spectrum of the produced gas mixture.

Table S1. Comparison of TOFs obtained by various catalysts for FA degradation.

Entry	Catalyst	TOF (h^{-1})
1	amine-functionalized SBA-15	355
2	mesoporous silica supported Pd-MnO_x	593
3	Pd silica nanosphere	85
4	mesoporous silica supported Pd	595
5	Pd@C-PS (photo-induced)	690
6	Pd@C-PS	378

Experimental

Materials and methods

All reagents were purchased from Sigma-Aldrich and used without further purification. MTMS and FA (85%) were purchased from Merck and Iranico companies, respectively. The Transmission and Scanning Electron Microscopies were carried out by TEM Philips EM 208S and SEM FEI Quanta 200. Pd determination was carried out on a flame atomic absorption spectroscopy (FAAS) (Shimadzu 105 model AA-680 atomic absorption spectrometer) with a hollow cathode lamp. The GC high-resolution gas chromatograph mass spectrometry was carried out using a Thermo

Scientific model. Fourier transform infrared spectroscopy (FT-IR) was used to characterise the different functional groups of the composite using a Jasco 6300 FTIR instrument in the range of 600–4000 cm^{-1}. HANDHELD RAMAN ANALYZER of RIGAKU (FIRSTGUARD model) was used for Raman spectroscopy. FlexPS equip (Specs) was used during the XPS measurements employing a monochromatic Al K_α (hν = 1486.6 eV) source, 100 W power and 10 kV voltage. The scans were acquired with a pass-energy of 20 eV, 20 scans per sample. The instrument work function was calibrated using C1s photoelectron. UV-Vis spectra were obtained by Agilent Cary 5000 UV-Vis spectrometer using quartz cuvettes with a path-length of 10 mm in H_2O as the solvent. Brunauer-Emmett-Teller analysis was done by Belsorp mini II instrument of Microtrac Bel Corp at 77 K after drying the sample under reduced pressure for 24 h. Origin 2019b was used to transform data to the graphs.
